# An Insight into All Tested Small Molecules against *Fusarium* *oxysporum* f. sp. *Albedinis*: A Comparative Review

**DOI:** 10.3390/molecules27092698

**Published:** 2022-04-22

**Authors:** Yassine Kaddouri, Redouane Benabbes, Sabir Ouahhoud, Magda Abdellattif, Belkheir Hammouti, Rachid Touzani

**Affiliations:** 1 Laboratory of Inorganic Chemistry, Department of Chemistry, University of Helsinki, P.O. Box 55, FI-00014 Helsinki, Finland; y.kaddouri@ump.ac.ma; 2 Laboratoire de Bioressources, Biotechnologie, Ethnopharmacologie et Santé (LBBES), Department of Biology, Faculty of Sciences, University Mohamed Premier, Oujda 11022, Morocco; red.bes72@gmail.com (R.B.); ouhaddouch@yahoo.fr (S.O.); 3 Chemistry Department, College of Sciences, Taif University, P.O. Box 11099, Taif 21944, Saudi Arabia; 4 Laboratory of Applied Chemistry and Environment (LCAE), Faculty of Sciences, University Mohammed Premier, Oujda 11022, Morocco; hammoutib@gmail.com

**Keywords:** pyrazole, imidazole, B-keto-enol, amino acid, quinoxaline, Bayoud, *Fusarium oxysporum* f. sp. *albedinis*

## Abstract

Bayoud disease affects date palms in North Africa and the Middle East, and many researchers have used various methods to fight it. One of those methods is the chemical use of synthetic compounds, which raises questions centred around the compounds and common features used to prepare targeted molecules. In this review, 100 compounds of tested small molecules, collected from 2002 to 2022 in Web of Sciences, were divided into ten different classes against the main cause of Bayoud disease pathogen *Fusarium oxysporum* f. sp. *albedinis* (F.o.a.) with structure–activity relationship (SAR) interpretations for pharmacophore site predictions as (δ^−^···δ^−^), where 12 compounds are the most efficient (one compound from each group). The compounds, i.e., (Z)-1-(1.5-Dimethyl-1*H*-pyrazole-3-yl)-3-hydroxy but-2-en-1-one **7**, (Z)-3-(phenyl)-1-(1,5-dimethyl-1*H*-pyrazole-3-yl)-3-hydroxyprop-2-en-1-one **23**, (Z)-1-(1,5-Dimethyl-1*H*-pyrazole-3-yl)-3-hydroxy-3-(pyridine-2-yl)prop-2-en-1-one **29**, and 2,3-bis-[(2-hydroxy-2-phenyl)ethenyl]-6-nitro-quinoxaline **61**, have antifungal pharmacophore sites (δ^−^···δ^−^) in common in N1---O4, whereas other compounds have only one δ^−^ pharmacophore site pushed by the donor effect of the substituents on the phenyl rings. This specificity interferes in the biological activity against F.o.a. Further understanding of mechanistic drug–target interactions on this subject is currently underway.

## 1. Introduction

Bayoud disease [1,2,3,4,5], caused by the telluric fungus pathogen *Fusarium oxysporum* f. sp. *albedinis* (F.o.a) [6,7,8,9], represents the leading dangerous agent of date palms cultivation, having killed more than 15 million Moroccan and Algerian date palm trees. Fungal infection causes significant implications, threatening date palms with high morbidity and mortality every year worldwide. Therefore, new antifungal inhibitors must be discovered urgently, especially those with new modes of action, low toxicity, and bioavailability, and are effective for responsive and drug-resistant fungi [10,11,12,13,14,15]. Due to their biological activity and chemical properties in recent years, fused heterocyclic compounds containing bridgehead nitrogen or oxygen donor atoms have drawn further interest. Indeed, several classes are reported in this review as pyrazole- and imidazole-based derivatives [16] presented in different biomolecules, such as histidine [17], histamine [18], and natural products [19]; this is an exciting building block [20]. Specifically, in recent decades, 4,5-diarylpyrazoles [21] and 2,5-diarylimidazoles [22] have gained interesting recognition as possible biomolecules in the field of drug development. Many biological and pharmacological properties are related to these structures [23]. βKeto-enol compounds [24,25,26,27] are found in many natural products as coumarin derivatives and play an important role in medicine and in the development of coordination chemistry as stable complexes. Imidazothiazole derivatives [28,29,30] are attractive nitrogen-containing heterocyclic ring-like histidine, biotin, nucleic acid, purine, etc., and have a broad spectrum of biological and pharmacological diverse activities.

Pyrazolic compounds [31] have established widespread potential biological activities, such as anti-inflammatory [32,33,34], antianxiety [35], antipyretic [36], antimicrobial [37,38,39,40], antiviral [41], antitumor [42,43,44], anticonvulsant [36,45,46,47], etc. Quinoxalines [48] are polyfunctionalized compounds with interesting biological activities, such as anti-human immunodeficiency virus (anti-HIV) and antidiabetic agents. Benzimidazole-1,2,3-triazole hybrid molecules [49] are hybrid compounds consisting of benzimidazole and 1,2,3-triazole, where both of them have a broad range of biological activities. *N*,*N*′-bipyrazole piperazine derivatives [50] are established as polypharmacological mixed ligands with several biological activities reported in the literature [51,52,53,54]. Meanwhile, Schiff base derivatives [53] have different biological functions, such as anti-inflammatory [55], antifungal [56], and antibacterial effects [57], and are commonly used as carriers of catalysts [58], optical chemical receptors [59], thermo-stable products [60], agents of metal complexion [61], inhibitors of corrosion [62], and stabilizers of polymers [63].

## 2. Pyrazole- and Imidazole-Based Derivatives

After some modifications, the agar diffusion approach is used for the antifungal analysis of pyrazole- and imidazole-based derivatives. In short, after isolation and preparation of the Fusarium fungus, the sterilized solution of the six compounds tested (**1**–**6**) in dimethyl sulfoxide (DMSO) is mixed with the potato dextrose agar (PDA) medium as an emulsifier at different concentrations using the method mentioned in the literature [16]. These compounds were synthesized by Takfaoui et al. using direct diarylation of pyrazoles and imidazoles with aryl halides, using palladium as the catalyst, DMAc as the solvent, and CsOAc as the base [64,65].

Using a non-linear regression algorithm curve of the concentration/percentage of inhibition, the half-maximal inhibitory concentration (IC_50_) was measured using Graphpad Prism software. DMSO-distilled water mixture was used as the negative control; no recognized antibiotic can specifically treat this infection.

The IC_50_ values are given in (Table 1) below. In the pyrazole derivatives, compound **4** (IC_50_ = 99.1 μg/mL) has the best fungus inhibition of all the tested compounds, where it contains p-C_6_H_4_ groups on the phenyl rings as an electron-donating character, and the high toxicity effect of the phenyl groups on the F.o.a. Furthermore, compound **1** (IC_50_ = 110.9 μg/mL), presenting m-CF_3_ groups on both phenyl rings, displays good activity close to that of compound **4**. However, the following compound is from the imidazole series (compound **5**) containing p-Cl groups on phenyl rings with an IC_50_ value equal to 114.7 μg/mL. The substitution of the phenyl rings by formyl (COH) groups (compound **6**) is highly unfavorable for inhibitory potency [16].

## 3. β-Keto-enol Derivatives

a β-Keto-enol Pyridine and Furan Derivatives

Using the agar diffusion process, we determined the in vitro antifungal ability of 11 compounds (**7**–**17**) against the pathogenic fungus (F.o.a). The synthetic route of the target compounds (**7**–**17**) was carried out following Claisen condensation under mild conditions [24,26,67,68,69,70,71,72,73,74]. Using the protocol described in the literature [27], the percentages of inhibition and semi-maximal inhibitory concentration (IC_50_) were measured and estimated using the inhibition percentage non-linear regression equation, while benomyl was used as a positive control (Table 2).

**Figure 1:**
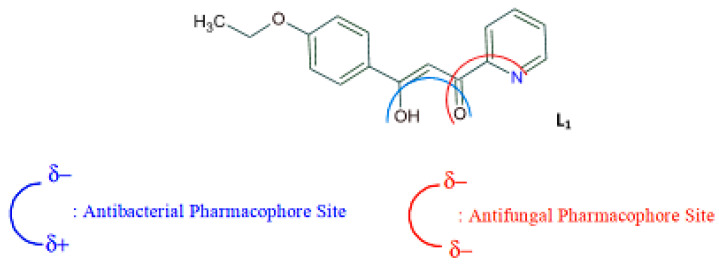
Antibacterial and antifungal pharmacophore sites for compound **7**.

As presented in Table 2, the fungal activity of **7** is very substantial, though it decreases slightly in the case of **10** because of ethoxy phenyl groups, which commonly have pharmacophore sites (δ^−^···δ^+^), as presented in Figure 1, due to their physicochemical properties and their ability to penetrate the envelope of fungal cells and enter their cellular place of action, thus displaying more excellent activity in [27].

b (Z)-3(3-bromophenyl)-1-(1,5-dimethyl-1*H*-pyrazol-3yl)-3-hydroxyprop-2-en-1-one derivatives

The agar diffusion technique was tested for in vitro antifungal function (ADT), where the literature reported the protocol details [7]. The optical density values were measured for each culture at 625 nm, and the inhibition percentage (%) is expressed as (D_0_ − Dx)/D_0_ × 100. D_0_ is the diameter of the mycelial growth of the culture witness, and Dx is the diameter of the mycelial growth (Table 3). The target biomolecules **18**–**23** based on βketo-enol and pyrazole entities and pyridine were prepared using a one-pot in situ condensation method, similar to the procedures in the literature [24].

As presented in Table 3, only compounds **22** and **23** reach values close to the standard (benomyl), as they belong to the same family. Such variations depend on the radical group attached to the fragment of pyrazole keto-enol, where compound **23** has a phenyl ring attached instead of the methyl group in compound **22**. In addition, numerous molecular improvements are currently being made to these compounds as antifungal candidates [25].

c β-Keto-enol pyrazolic compounds

The in vitro antifungal potential of ten prepared βKeto-enol pyrazolic compounds against the pathogen F.o.a was determined by the agar diffusion technique reported in the literature [26], and the half-maximal inhibitory concentration (IC_50_) was determined using a non-linear regression algorithm of the concentration-inhibition percentage graph, with benomyl used as a positive control. In addition, the target biomolecules **24**–**30** based on βketo-enol and pyrazole entities were prepared by a one-pot in situ condensation method, which is similar to the procedures given in the literature [24].

On the other hand, most of these molecules demonstrate potent antifungal action against F.o.a, as seen in Table 4. These were based on the structure–activity relationships (S.A.R.s). Where a stimulating effect is exerted against F.o.a of the substitution pattern, we found compound **28** in the 3-thiophene group. In contrast, compound **30** with the 2-naphthalene group led the same inhibition percentage of 94% as the benomyl fungicide, while the best antifungal activity was found for compound **29** containing the 2-pyridine group IC_50_ of 60.84 μg/m. The existence of the R substituent should be further exploited [8] to evaluate the S.A.R.s for this novel class of antifungal agents.

## 4. Imidazothiazole Derivatives

The synthesis of various types of imidazothiazoles **31**–**35** is potentially helpful for developing biologically active heterocycles. The synthetic methods are practical and straightforward and are conceivably applicable to analogous heterocyclic systems possessing nitrogen and sulfur [30,75,76,77,78,79,80,81,82]. The antifungal action of five imidazothiazole derivatives **31**–**35** is carried out on an F.o.a using the concentrations C_1_, C_2_, C_3_, C_4_, and C_5_ as 5.0, 1.0, 0.2, 0.05, and 0.01 mg/mL, respectively. Each compound was prepared at various concentrations in the potato dextrose agar (PDA) before the fungus was cultured using the protocol described in the literature [28]. The IC_50_ was calculated using the linear regression equation between the normal logarithm concentrations and growth inhibition percentages.

From Table 5, the antifungal test of the five imidazothiazole derivatives tested against F.o.a. at five different concentrations acted differently, while all the molecules showed interesting results. Indeed, the best antifungal activity is found for compound **33** due to three methyl substituents on the ortho and para positions of the phenyl ring with IC_50_ not exceeding 20.00 μg/mL [28].

## 5. Pyrazolic Compounds

Monopyrazolic heterocyclic compounds **36**–**55** were prepared in excellent yields by condensing one equivalent of hydroxymethylpyrazole with one equivalent of primary amines [83,84,85]. The antifungal behavior, as defined in the literature, was calculated by the agar diffusion technique [31], with the linear regression equation between the normal logarithm of the concentrations and the growth inhibition percentages calculated at the half-maximal inhibitory concentration (IC_50_).

The pyrazolic derivatives **50**, **51**, and **53**–**55** were screened in vitro for their antifungal potential against F.o.a and collected in Table 6, where compounds **50** and **55** showed an excellent efficacy of IC_50_ = 86 μM and 168 μM, respectively, arguably due to the presence of the two phenyl moieties. Due to the (-Br) group, which is an essential source of electronegativity, compound 53 showed a moderate potential with an IC_50_ = 284 μM. The two other pyrazoles tested demonstrated low antifungal function [31].

## 6. Quinoxalines

A variety of 2,3-bifunctionalized quinoxalines (**56**–**61**) have been prepared by the condensation of 1,6-disubstituted-hexan-1,3,4,6-tetraones with o-phenylenediamine, (R,R)-1,2-diaminocyclohexane, and p-nitro-o-phenylenediamine [86,87,88]. The antifungal activity of six prepared quinoxaline compounds’ antifungal activity was measured against F.o.a, as described in the method in the literature [48].

Based on Table 7, the most effective inhibitor is nitroquinoxaline **61**, which produces 51% inhibition of the growth of Fusarium at a concentration of 72 mg/L due to its small nitro groups that disturb the cell membrane, with some intracellular target and electron-withdrawing solid group. At the same time, compounds **56**, **60**, and **59** are less effective but produce appreciable growth inhibition at comparable concentrations [48].

## 7. Benzimidazole-1,2,3-triazole Hybrid Molecules

A series of hybrid molecules **62**–**69** was prepared by condensing 4-(trimethylsilylethynyl)benzaldehyde with substituted o-phenylenediamines. These, in turn, were reacted with 2-(azidomethoxy)ethyl acetate in a Cu alkyne–azide cycloaddition (CuAAC) to generate the 1,2,3-triazole pharmacophore under microwave assistance [89,90,91,92].

The eight new benzimidazole-1,2,3-triazole hybrid molecules were tested against F.o.a using the method described in the literature [49], and their linear growth and sporulation inhibitory rates are presented in Table 8.

Based on Table 8, all compounds were tested at a 20 mg/mL concentration, with Pelt, a systemic fungicide and benzimidazole precursor (70% of methyl thiophanate), as the positive control. Compound **66** shows a significantly increased rate with (17.01 and 30.62%) (*p* < 0.05) against F.o.a, which uniquely holds a CF_3_ group fixed to the benzimidazole core, a lipophilic group known to modulate absorption and metabolism, and may explain the enhanced activity [49].

## 8. *N*,*N*′-Bipyrazole Piperazine Derivatives

Novel bipyrazoles **70**–**73** possessing piperazine or a mimed piperazine ring spacer were prepared in a one-step reaction in excellent yields. First, it condensed two hydroxymethylpyrazole derivatives with one equivalent of cyclic and acyclic piperazine [93,94,95,96].

As stated in the literature, in vitro antibacterial and antifungal activity is tested by the agar diffusion technique [50] using pathogenic strains of F.o.a. In contrast, streptomycin was used in the antibacterial assay as a reference compound for quality reasons. Therefore, the minimal concentration of inhibition (M.I.C.) is the lowest concentration of the tested compound that has inhibited the development of the micro-organism.

As presented in Table 9, four tested compounds showed differential anti-proliferative activity against F.o.a, as the best M.I.C. value was found for compound **71** of 5 μg/mL. These results are explained by the piperazine ring spacer and the carboxylate moiety at the three-position of the pyrazole rings that considerably increases the antifungal activity [50].

## 9. Bipyrazolic Tripodal Derivatives

A series of novel bipyrazolic tripodal derivatives **74**–**81** was prepared in one step, with good and excellent yields. Then, one equivalent of the appropriate amine derivatives was added to a solution of two equivalents of the substituted hydroxymethylpyrazole in acetonitrile, and the mixture was continued under stirring at room temperature for 4–5 days. Finally, the crude material was evaporated, washed with water and CH_2_Cl_2_, and purified by silica gel column flash chromatography to give the target product **74**–**81** [52].

The eight compounds containing bipyrazolic tripod derivatives are tested in vitro for their efficacy against *Fusarium oxysporum* f. Isolated from a date palm with vascular fusariosis, F.o.a was used as the protocol described in the literature [52]. The minimum inhibition concentration (M.I.C.) is the lowest dose of the compound that can inhibit micro-organism development.

From data in Table 10, the presence of the methyl as electron donor groups on the pyrazole rings increased the antifungal activity for compounds **74**, **76**, **78**, and **80**, but has a counter effect on the phenyl ring, e.g., in the case of compounds **80** and **81** which have M.I.C. values of 40 and 80 μg/mL, respectively. Additionally, nitro substituent as an electron-withdrawing group for compound **79** increased its effect compared with compound **77** [52].

## 10. Schiff Base Derivatives

Twelve new Schiff base derivatives are prepared using the condensation reaction of different amino-substituted compounds (such as aniline, pyridine-2-amine, o-toluidine, 2-nitrobenzenamine, 4-aminophenol, and 3-aminopropanol) and substituted aldehydes (such as nicotinaldehyde, o,m,p-nitrobenzaldehyde, and picolinaldehyde) in ethanol with acetic acid as a catalyst [53].

The agar diffusion technique against *Fusarium oxysporum f* evaluated the in vitro antifungal activities of all the new Schiff base derivative compounds, including F.o.a fungus, as described earlier [53]. In the presence of a concentration of the tested compound over the mycelium diameter of the reference culture multiplied by 100, it is found that the inhibition proportion of a molecule is proportional to the ratio of the mycelium diameter of the culture. Therefore, the minimal concentration of inhibition (M.I.C.) is the lowest dose of the compound, which inhibited the growth of the microorganism when the mixture (DMSO/EtOH + distilled water) is used as a negative control without any standard reference drug.

On the contrary, based on their M.I.C. values in Table 11, the in vitro antifungal assay findings showed that most of the screened ligands exhibited high to moderate activity against F.o.a. The maximum activity was 0.02 µg/mL, shown by compound **84**, followed by compounds **87**, **88**, and **93** with M.I.C. values equal to 0.04 µg/mL, while compound **83** showed the most negligible M.I.C. value of 0.9 µg/mL. Other products also have numerous activities, with M.I.C.s varying from 0.08 µg/mL for compound **92** to 0.30 µg/mL for compound **86**. Comparing both the structures of **83** and **84**, it can be inferred that the presence at the ortho position of the phenyl ring of a strong electron-withdrawing group, such as nitro moiety (NO_2_), is very appropriate for increasing antifungal efficiency; the presence of an electron donation group, such as methyl moiety (CH_3_) for antifungal action, is unfavorable in the period. Unfortunately, though, the correct variables that influence the antifungal ability of these derivatives are difficult to ascertain with these early investigations. Further investigations using other models and techniques are essential for this [53].

## 11. Amino Acids Pyrazole Compounds

The functional pyrazolyl derivatives **94**–**100** were prepared by condensing two equivalents of (3,5-dimethyl-1H-pyrazole-1-yl)methanol with one equivalent of amino acid ester hydrochloride derivatives (commercially available) in anhydrous solvents. All reactions were carried out at room temperature under stirring conditions for 4 to 6 days in an inert atmosphere [42,97,98,99,100,101,102,103,104,105,106].

The activities of the pyrazole compound amino acids and the agar techniques determined **94**–**100**. The yeast of the F.o.a was isolated from a date palm touched by the vascular Fusarium prepared in a PDA medium at 37 g/L [54].

Based on Table 12, compared to blank culture, the inhibition rates of F.o.a development ranged from 0 to 480 mg/L for ester hydrochloride amino acids or their tripodal pyrazolic homologs. Inhibition activity against the growth of F.o.a. was shown by the various compounds studied, except **94** and **95**. However, the rate of this inhibition changes from one molecule to another. Compound **98** has the best antifungal activity due to methyl substituents as electron donor groups in methyl alaninate (alanine ester) as the amino acid; these products’ structural and electronic diversity affected their biological activities. Further developments on this subject are currently in progress in order to understand their mechanistic interactions [54].

## 12. Comparison Using Structure–Activity Relationship

**Figure 2:**
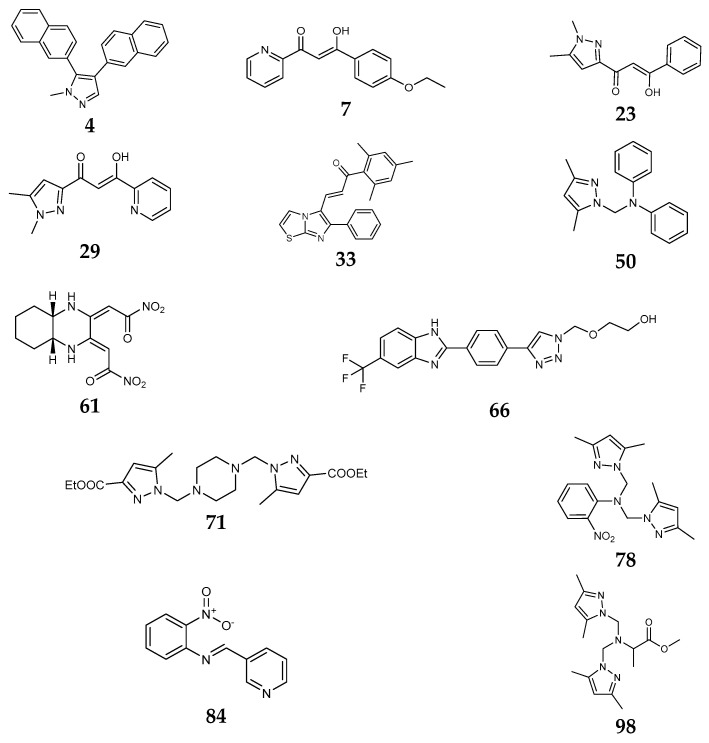
Chemical structure of the best active compounds from the group.

To understand this structure–activity relationship and the modes of action of these new biologically active molecules, we can carry out a theoretical study with bioinformatics molecular modeling (DFT, Docking, and ADME-Tox studies) after studying the mechanism of the reaction using conceptual DFT [107,108]. As a result, we obtained various prospective targeted drugs as inhibitors for Bayoud disease (Figure 2).

As presented in Figure 2, compounds **7**, **23**, **29**, and **61** have the antifungal pharmacophore sites (δ^−^···δ^−^) in common in N_1_---O_4_, whereas other compounds have only one δ^−^ pharmacophore site pushed by the donor effect of the substituents on the phenyl rings; this specificity interferes in the biological activity against F.o.a.

## 13. Conclusions

This review uses 100 compounds of tested small molecules divided into ten classes against *Fusarium oxysporum* f. sp. *albedinis* (F.o.a). First, compound **4** (IC_50_ = 99.1 μg/mL) has the best fungus inhibition over all the pyrazole and imidazole derivatives, containing electron-donating character as para phenyl substituents. Furthermore, it is displays high toxicity in the phenyl groups on the F.o.a. Second, from βketo-enol derivatives, compounds **7**, **23**, **29**, and **61** have the antifungal pharmacophore sites (δ^−^···δ^−^) in common in N1---O4, whereas other compounds have only one δ^−^ pharmacophore site pushed by the donor effect of the substituents on the phenyl rings; this specificity interferes in the biological activity against F.o.a. Moreover, these products’ structural and electronic diversity can affect their biological activities. Further developments on this subject are currently in progress to better understand their mechanistic interactions.

## Figures and Tables

**Table 1:** IC_50_ values of the tested pyrazole- and imidazole-based derivatives tested against F.o.a.

ID.	Structure	IC_50_	
		μg/mL	μM
**1**	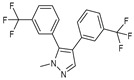	110.9	299.4
**2**	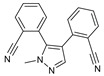	153.2	538.8
**3**	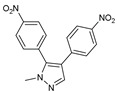	165.1	509.1
**4**	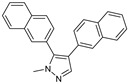	99.1	256.4
**5**	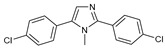	114.7	378.3
**6**	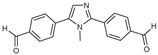	194.5	667.1

Compared with literary works, we found that the pyrazole skeleton and its derivatives exhibited excellent inhibitory activity against *Fusarium oxysporum* [66].

**Table 2:** IC_50_ values of the tested βketo-enol pyridine and furan derivatives against F.o.a.

ID	Structure	IC_50_	
		μg/mL	μM
**7**	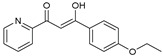	12.83	
**8**	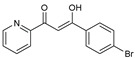	NS	NS
**9**	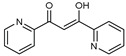	NS	NS
**10**	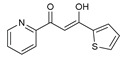	17	
**11**	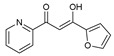	36	
**12**	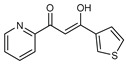	-	-
**13**	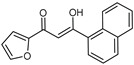	-	-
**14**	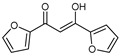	-	-
**15**	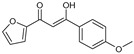	-	-
**16**	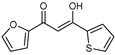	-	-
**17**	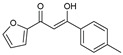	-	-

**Table 3:** Volume is withdrawn, a diameter of the strain and inhibition percentages of the tested (Z)-3(3-bromophenyl)-1-(1,5-dimethyl-1*H*-pyrazole-3yl)-3-hydroxyprop-2-en-1-one derivatives **18**–**23** against F.o.a.

ID	Structure	Volume Is Withdrawn (μL)	Diameter of the Strain in the Presence of the Drug (cm)	Inhibition (%)
**18**	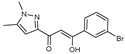	50 200 500	5.0 3.8 2.7	0 24 46
**19**	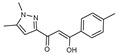	50 200 500	5.0 3.5 2.3	0 30 54
**20**	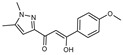	50 200 500	5.0 3.6 2.5	0 28 50
**21**	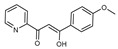	50 200 500	5.0 3.8 3.2	0 24 36
**22**	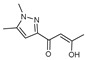	50 200 500	1.2 0.9 0.5	76 82 90
**23**	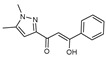	50 200 500	2.0 1.3 0.2	60 74 96
**Benomyl**	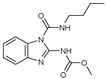	50 200 500	2.3 1.1 0.3	54 78 94

**Table 4:** IC_50_ values of the tested βketo-enol pyrazolic derivatives against F.o.a.

ID	Structure	IC50	
		μg/mL	μM
**24**	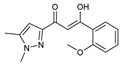	-	-
**25**	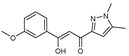	260.74	71
**26**	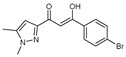	-	-
**27**	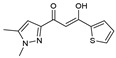	-	-
**28**	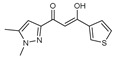	193.31	48.00
**29**	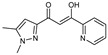	60.84	14.80
**30**	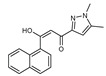	181.30	53.00

**Table 5:** IC_50_ values of the tested imidazothiazole derivatives against F.o.a.

ID	Structure	IC_50_ (μg/mL)
**31**	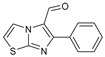	50.00
**32**	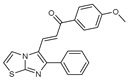	70.00
**33**	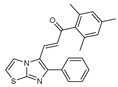	20.00
**34**	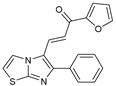	60.00
**35**	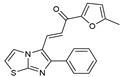	50.00

**Table 6:** IC_50_ values of the tested pyrazolic compounds against F.o.a.

ID	Structure	IC_50_ (μM)
**36**	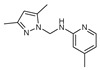	-
**37**	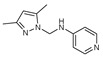	751
**38**	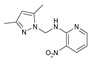	2507
**39**	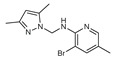	406
**40**	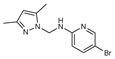	398
**41**	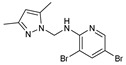	333
**42**	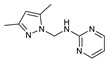	2755
**43**	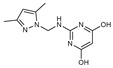	2550
**44**	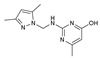	2486
**45**	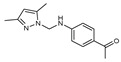	2614
**46**	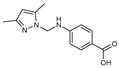	1223
**47**	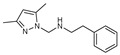	697
**48**	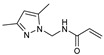	2856
**49**	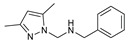	2322
**50**	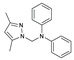	86
**51**	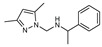	662
**52**	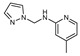	2592
**53**	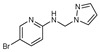	284
**54**	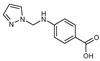	-
**55**	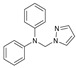	168

**Table 7:** Percent growth inhibition at different concentrations for quinoxaline compounds tested against F.o.a.

ID	Structure	Percent Growth Inhibition (Concentration, mg/L)		
		C1	C2	C3
**56**	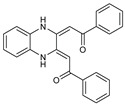	9 (20)	7 (40)	22 (80)
**57**	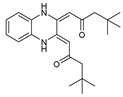	9 (60)	15 (120)	15 (180)
**58**	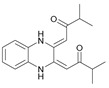	17 (60)	17 (120)	19 (180)
**59**	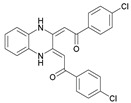	21	32	35 (180)
**60**	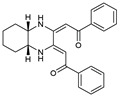	15 (34)	31 (67)	33 (134)
**61**	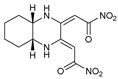	29 (18)	31 (36)	51 (72)

**Table 8:** Linear growth and inhibitory sporulation rates of benzimidazole-1,2,3-triazole hybrid molecules tested against F.o.a.

ID	Structure	Linear Growth-Inhibitory Rates (%)	Sporulation Inhibitory Rates (%)
**62**	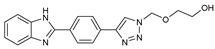	3.02 ± 0.96	−5.85 ± 0.04
**63**	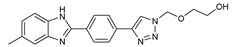	−1.59 ± 0.05	16.36 ± 0.2
**64**	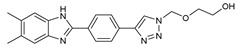	2.7 ± 0.16	−34.79 ± 0.72
**65**	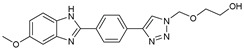	−0.16 ± 0.02	21.94 ± 0.26
**66**	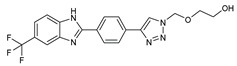	17.01 ± 0.96	30.62 ± 0.5
**67**	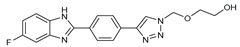	2.3 ± 0.29	−77.59 ± 2.64
**68**	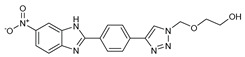	−1.41 ± 0.3	−61.05 ± 1.34
**69**	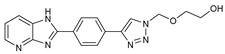	−14 ± 0.05	−48.72 ± 2.35

**Table 9:** M.I.C. values of *N*,*N*′-bipyrazole piperazine derivatives tested against F.o.a.

ID	Structure	M.I.C.	
		μg/mL	μM
**70**	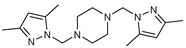	10	33.06
**71**	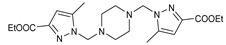	5	11.94
**72**	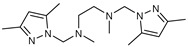	10	32.85
**73**	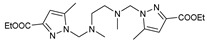	20	47.56

**Table 10:** M.I.C. values of bipyrazolic tripodal compounds tested against F.o.a.

ID	Structure	M.I.C.	
		μg/mL	μM
**74**	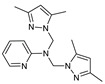	2.5	8.05
**75**	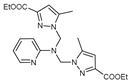	5	11.73
**76**	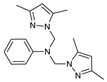	2.5	8.08
**77**	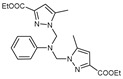	40	94.7
**78**	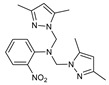	2.5	7.05
**79**	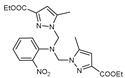	5	10.63
**80**	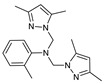	40	123.84
**81**	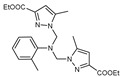	80	182.14

**Table 11:** M.I.C. values of Schiff base derivatives compounds tested against F.o.a.

ID	Structure	MIC (μg/mL)
**82**	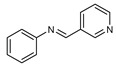	0.10
**83**	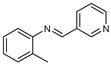	0.90
**84**	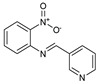	0.02
**85**	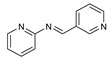	0.25
**86**	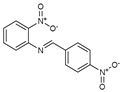	0.30
**87**	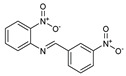	0.04
**88**	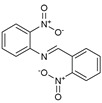	0.04
**89**	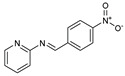	0.12
**90**	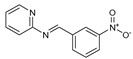	0.25
**91**	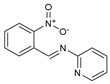	0.20
**92**	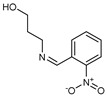	0.08
**93**	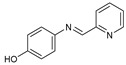	0.04

**Table 12:** MIC values of amino acids pyrazole compound tested against F.o.a.

ID	Structure	MIC (mg/L)
**94**	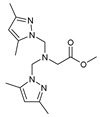	-
**95**	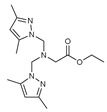	-
**96**	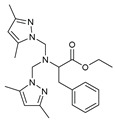	17
**97**	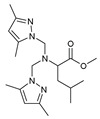	15
**98**	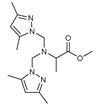	0.3
**99**	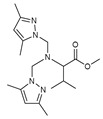	10
**100**	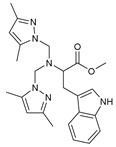	0.5
